# Caregiver-related predictors of thermal burn injuries among Iranian children: A case-control study

**DOI:** 10.1371/journal.pone.0170982

**Published:** 2017-02-02

**Authors:** Homayoun Sadeghi-Bazargani, Reza Mohammadi, Erfan Ayubi, Amir Almasi-Hashiani, Reza Pakzad, Mark J. M. Sullman, Saeid Safiri

**Affiliations:** 1 Road Traffic Injury Research Center, Department of Statistics & Epidemiology, Tabriz University of Medical Sciences, Tabriz, Iran; 2 WHO Collaborating Center on Community Safety Promotion, Karolinska Institute, Stockholm, Sweden; 3 Department of Epidemiology, School of Public Health, Shahid Beheshti University of Medical Sciences, Tehran, Iran; 4 Department of Epidemiology and Reproductive Health, Reproductive Epidemiology Research Center, Royan Institute for Reproductive Biomedicine, ACECR, Tehran, Iran; 5 Student Research Committee, Ilam University of Medical Sciences, Ilam, Iran; 6 Driving Research Group, Cranfield University, Bedfordshire, United Kingdom; 7 Managerial Epidemiology Research Center, Department of Public Health, School of Nursing and Midwifery, Maragheh University of Medical Sciences, Maragheh, Iran; 8 Department of Epidemiology and Biostatistics, School of Public Health, Tehran University of Medical Sciences, Tehran, Iran; Centre for Injury Prevention and Research, Bangladesh (CIPRB) & Örebro University, Sweden, BANGLADESH

## Abstract

**Purpose:**

Burns are a common and preventable cause of injury in children. The aim of this study was to investigate child and caregiver characteristics which may predict childhood burn injuries among Iranian children and to examine whether confounding exists among these predictors.

**Methods:**

A hospital based case-control study was conducted using 281 burn victims and 273 hospital-based controls, which were matched by age, gender and place of residence (rural/urban). The characteristics of the children and their caregivers were analyzed using crude and adjusted models to test whether these were predictors of childhood burn injuries.

**Results:**

The age of the caregiver was significantly lower for burn victims than for the controls (*P<*0.05). Further, the amount of time the caregiver spent outdoors with the child and their economic status had a significant positive association with the odds of a burn injury (*P<*0.05). A multivariate logistic regression found that Type A behaviour among caregivers was independently associated with the child's odds of suffering a burn injury (OR = 1.12, 95% CI: 1.04–1.21). The research also found that children with ADHD (*Inattentive subscale*: Crude OR = 2.14, 95% CI: 1.16–3.95, Adjusted OR = 5.65, 95% CI: 2.53–12.61; *Hyperactive subscale*: Crude OR = 1.73, 95% CI: 1.23–2.41, Adjusted OR = 2.53, 95% CI: 1.65–3.87) also had increased odds of suffering a burn injury. However, several variables were identified as possible negative confounder variables, as the associations were stronger in the multivariate model than in the crude models.

**Conclusion:**

The caregiver's characteristics which were predictors of burn injuries among Iranian children were: being younger, high socio-economic status, Type A behavioural pattern and spending more time outdoors. In addition, the relationship between a child's ADHD scores and the odds of a burn injury may be negatively confounded by the caregivers predictor variables.

## Introduction

Unintentional injuries are a health concern in every country around the world and result in over 5 million deaths per year, or 16,000 deaths per day [1]. According to the World Health Organization’s Global Burden of Disease Study, unintentional injuries accounted for over 3.9 million deaths in 2004. Furthermore, five of the 15 leading causes of death among persons 15–29 years of age are accidental, including: road traffic injuries, drowning, burns, poisoning, and falls [1]. A worldwide increase in prevention efforts and early intervention programmes have substantially reduced the burden of unintentional injuries as a public health concern [2]. However, a systematic review has shown that burn injuries remain an important public health issue in the East Mediterranean Region (EMR) [3] and also in Iran [4, 5].

According to WHO, the incidence rate of burns in low- and middle-income countries is 1.3 per 100,000 people, compared to 0.14 per 100,000 people in high-income countries. In the EMR this incidence is 29 per 100,000 people per year, which is significantly higher than the lowest incidence in the Americas (8 per 100,000). Furthermore, over 95% of fatal fire-related burn injuries occur in low- and middle-income countries [3, 6, 7].

In Iran, which is a middle income country, the National Burden of Disease Study in 2003 showed that burn injuries were the 13^th^ most frequent cause of injury in the general population, and the 7^th^ most common among children aged 5–14 years old [8]. While childhood burns account for 3.5–10% of all burns worldwide [1, 9], childhood burns are a major concern in Iran and comprise up to 38% of all burns in Iran [10, 11]. Despite being a preventable cause of injury among children, burns still cause significant morbidity and mortality in Iran [12] and are therefore an important public health concern.

As burn patients need special care and equipment, in addition to highly trained staff, the treatment of burns is sophisticated, expensive, and time consuming. Therefore, prevention is much more desirable than treatment [12]. Due to cultural and environmental differences, the predictors of burn injuries should be investigated in each country, to allow for the identification and implementation of preventative measures. The epidemiology of childhood burn injuries in Iran has been investigated by several studies [12–14]. However, surprisingly the individual predictors of these injuries, including psycho-social predictors, have rarely been researched [15]. Unlike burns in adulthood, the predictors of childhood burns are not largely limited to the individual alone, the characteristics (e.g., personality type, age, education, sex, economic status and time spent outdoors) of the caregiver (including the mother or anyone else who supervises the children) must also be taken into consideration.

Therefore, the aim of the current study was to investigate caregiver-related predictors of childhood burn injuries in Iran and to determine whether child and caregiver predictors were confounded.

## Methods

A case-control study was undertaken at Sina University General Hospital, which is located in the city of Tabriz. Tabriz is the capital city of the East Azerbaijan province in the northwest of Iran, and has a population of around four million people. The study was conducted over a period of 12 months, from 2009 to 2010. The Hospital receives burn injury cases from 19 districts in the province. In order to minimise bias, participants’ age, gender and place of residence (urban/rural) were frequency matched for the case and control groups.

WHO denotes a “burn” as an injury to the skin or other organic tissue primarily caused by heat, but can also be because of radiation, radioactivity, electricity, friction or contact with chemicals, or respiratory damage resulting from smoke inhalation [16]. These injury mechanisms all fall into ICD 10 (International Classification of Diseases) chapter 19: T20-T32 and chapter 20: X00-X19 coding categories. Exposure to electric current (W85-W87) can also be included, if this led to any injuries in the T20-T32 coding categories. However, in order to meet the objectives of this study, only thermal burns were investigated.

The core potential key variables, to be investigated, were selected based upon the available literature, in particular the WHO report on child injuries, Forjuoh et al. and the researchers own experience [10, 17–20]. Using the Haddon’s matrix as a framework for conceptualizing the potential predictors, the variables were extended via discussions using an expert panel which included: the researchers, an experienced burn surgeon, a psychiatrist and an epidemiologist specialising in injuries [21].

The data was collected using a questionnaire administered by an interviewer, except in a small number of cases where the caregiver completed the questionnaire themselves, which were checked for completeness by the interviewer. For the purpose of this study four interviewers were selected from the hospital’s registered medical staff, including three registered medical experts. These staff had completed two years of academic education in the medical registry before being engaged by the hospital. In addition, these staff members participated in a short training session and supervised data collection during the pilot study to ensure interviewer consistency. The reliability and validity of data collection was addressed by asking each interviewer to conduct the same number of interviews with cases and the matched controls. This method was conducted in order to reduce information bias through the comparable accuracy principle in case–control studies. As this study involved assessing burn injuries among children, either their parents or caregivers were interviewed for about 30 minutes.

Childhood Attention Deficit Hyperactivity Disorder was assessed using the ADHD-Rating Scale [22] and the ADHD ratio was calculated per ten score increment.

As this study involved assessing socio-economic status in a low and middle-income country, the consumption expenditure method was used to measure socio-economic status. The variables measured in the present study included the ability to provide: clothing, food, jewellery, furniture, travel and education costs. Principal Component Analysis was used to combine the economic status variables into a single weighted variable, which was then transformed into quintiles. All stages of the analysis involved determining the suitable scales of variables and model building, as recommended by Jewell in Statistics For Epidemiology [23].

### Cases

The cases were comprised of 281 injured children of both sexes and all social classes who were hospitalized in the Sina University Hospital. Only those with accidental acute thermal injury which met the inclusion criteria were included in the case group, after consent to participate was obtained. Data from participants who died before the interview and assessment were not included in the study. Willing patients who met the following inclusion and exclusion criteria were enrolled in the case group.

### Inclusion criteria

1. Patients living in the East Azerbaijan Province for at least the previous four weeks.
2. Patients with the appearance of burn injuries, such as: scalds, flame burns and contact burns that happened either indoors or outdoors.

### Exclusion criteria

1. Burn injuries that happened outside the study region
2. Patients with intentional burns (e.g., child’s play) and self-immolation
3. Patients with non-thermal burn injuries, such chemical burns and cold burns
4. Patients burnt in catastrophes or disasters.
5. Patients with burns related to child abuse
6. Patients that were not hospitalized
7. Burn injuries with inadequate information

### Controls

The control group consisted of 273 hospitalized patients from the Tabriz Pediatric Hospital, with a similar referral pattern. All subjects were chosen as per the selection principles for case–control studies [24, 25]. The general pediatric clinic that frequently received patients from regions nearby was excluded in order to meet the common population source principle. In addition, the control selection process was undertaken on a case by case basis. For example, to ensure the independence of exposure while minimizing recall bias, children under long-term intensive care for diseases that severely affected their life style were excluded. For example, a child who has been undergoing intensive oncologic treatment for the past 6 months will have different rates of exposures to activities such as playing outdoors or watching TV, when compared to the ideal control population and asking them about the situation prior to their condition would not produce reliable information. All possible control measures were undertaken to reduce the chances of both selection and recall bias.

Patients who fell within the following inclusion and exclusion criteria, and were willing to participate in the study, were included in the control group.

### Inclusion criteria

1. Patients without a history of burn-related injuries who were residing in the study area for the four weeks immediately prior to admission.
2. Patients admitted to Tabriz Pediatric Hospital for other reasons.
3. Patients of similar age, gender and place of residence (rural vs urban) as those in the case group.

### Exclusion criteria

1. Patients suffering from diseases that have lasted more than three months (chronic medical conditions) or other major injuries
2. Patients that were not hospitalized.

### Statistical analysis

Firstly the data were analysed to determine whether the items in the Type A questionnaire [26] was suitable for factor analysis. This found that all correlation coefficients ranged from 0.3 to 0.8, the Kaiser-Meyer-Olkin test was >0.7, Bartlett’s test was significant (*p*-value<0.05) and the communalities (>0.5) were acceptable for factor analysing. Factor analysis was undertaken for all subjects using principal component analysis (PCA) in three steps: 1) extraction of the factors; 2) rotation of the factors to aid interpretation; and 3) naming and interpretation of the factors, based on the factor loadings. Factors with eigenvalues greater than 1 were selected and Varimax rotations were used. Only factor loadings of 0.4 and above were considered to determine which variables comprised each factor. The robustness of the exploratory factor analysis (EFA) was then assessed using confirmatory factor analysis (CFA), which tests the construct validity of the factor solution. The goodness-of-fit statistics used were: χ2 statistic, comparative fit index (CFI), goodness of fit index (GFI), incremental fit index (IFI), McDonald fit index (MFI), root mean square error of approximation (RMSEA), root mean square residual (RMSR), and the standardized root mean square residual (SRMR).

Quantitative and qualitative variables for cases and controls were presented as mean (SD) and frequency (%), respectively.

Binary logistic regression models were used to relate the independent variables to subject status (case/control) and the odds ratios (OR) were calculated with 95% confidence intervals.

Both univariate and multivariate (adjusted by sex, age and economic status of the child, as well as the age and economic status of the caregivers) models were constructed. Stata (Version 11) and Multivariate Software (EQS) (Version 6.1) were used for all data analyses. Two tailed p-values of <0.05 were used as the criteria for statistical significance.

### Ethical issues

All protocols were approved by the regional ethics committee of Tabriz University of Medical Sciences and the research was carried out in compliance with the Helsinki Declaration. Data from burn injuries was only collected with the written consent of parents and the complementary assent of children older than 7 years old. The ethics committees approved consent procedure. All of the written consents were documented in research room.

## Results

**Fig 1:**
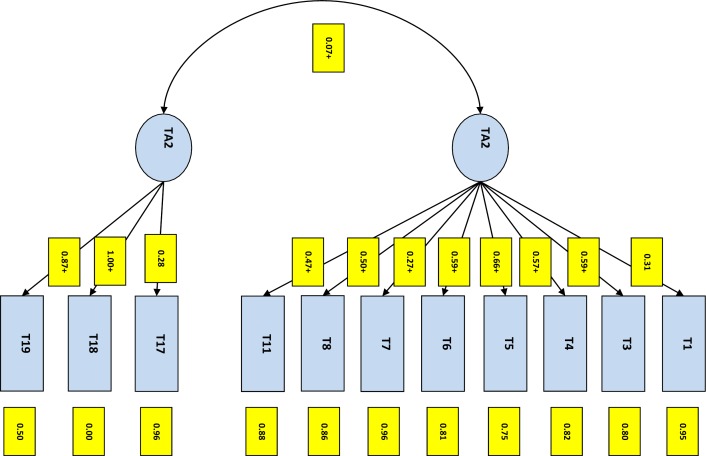
The two factor uncorrelated model of Type A personality, as suggested by Nadjarian et al. [26].

Firstly, the 11 Type A items satisfied the entrance criteria into the PCA model. This revealed two factors with Eigenvalues ≥ 1, which accounted for 26.2% and 20.5% of the variance, respectively. CFA was then undertaken to validate this factor structure, with the *fit* statistics of the hypothesized models presented in Table 1. The two-factor model met fit criteria (CFI = 0.89, RMSEA = 0.08), although χ2 was significant, which is due to the large sample size. Fig 1 illustrates the hypothesized two factor model. Consequently, the two subscales (*TA1* and *TA2*) were used for assessing Type A behaviour among caregivers [26].

**Table 1:** Summary of CFA fit statistics.

Model fit	χ2	*df*	*p*-value	CFI	GFI	IFI	MFI	RMSEA	RMSR	SRMR
**2-factor model**	201.54	43	<0.001	0.89	0.93	0.89	0.85	0.08	0.09	0.09

CFI, comparative fit index; GFI, goodness of fit index; IFI, incremental fit index; MFI, McDonald fit index; RMSEA, root mean square error of approximation, RMSR, root mean square residual; SRMR, standardized root mean square residual.

The child and caregiver characteristics of the 281 case patients and 273 control subjects are presented in Table 2. The age of participants ranged from 6 months to 12 years. Subjects from both groups had similar demographic characteristics, such as the child’s sex and the caregivers’ level of education (*P* >0.05). However, the mean age of the caregivers in the control group was higher than the case group (*P<*0.05). The covariate variables that differentiated case from control subjects in the univariate analyses were: childhood attention deficit hyperactivity disorder (ADHD), having burn scars, time spent outdoors with the caregiver, the caregiver's Type A behaviour type and high socio-economic status (*P<*0.05). The univariate OR (95% CI) for TA2 (Type A subscale) and *Inattention* and *Hyperactivity* (subscales of ADHD) were 1.12 (1.04–1.21), 2.14 (1.16–3.95) and 1.73 (1.23–2.41), respectively. A multivariate analysis to investigate the relationships the caregiver's personality type and childhood ADHD subscales had with childhood burn injuries was undertaken, including potential confounding variables (i.e., variables that had p-values < 0.20 in the univariable analysis). This found that the covariates in the model were negative confounders, with ORs (95% CI) of 5.65 (2.53–12.61) and 2.53 (1.65–3.87) for Inattention and Hyperactivity subscales, respectively (Table 3).

**Table 2:** Univariate analysis of the child and caregiver variables.

Child variables	Control (n = 273)	Case (n = 281)	OR (95%CI)	*p*-value ^a^
**Age (year)**	5.66 (0.23)	3.27 (0.14)	0.79 (0.75–0.84)	<0.001
**Sex (male)**	127 (47.04)	109 (39.07)	0.72 (0.51–1.01)	0.06
**Having musculoskeletal disorders (yes)**	13 (4.81)	9 (3.22)	1.51 (0.0.63–3.61)	0.34
**Total ADHD**				
*Inattentive*	2.30 (0.15)	2.89 (0.17)	2.14 (1.16–3.95)	0.014
*Hyperactive*	6.51 (0.30)	7.91 (0.30)	1.73 (1.23–2.41)	0.001
**Having epilepsy (yes)**	18 (6.64)	9 (3.25)	0.47 (0.21–1.07)	0.07
**Having burns history in last year (yes)**	18 (6.64)	12 (4.29)	1.58 (0.75–3.36)	0.22
**Having burns history in family (yes)**	72 (26.47)	68 (24.37)	1.11 (0.76–1.63)	0.57
**Having burns scar (yes)**	48 (17.71)	70 (25.83)	0.61 (0.40–0.93)	0.023
**Having falling history (yes)**	9 (3.33)	12 (430)	0.76 (0.31–1.85)	0.55
**Having inflammable clothes in the home**				
Very low	31 (11.40)	21 (7.61)	References	
Low	159 (58.46)	135 (48.91)	1.25 (0.68–2.28)	0.46
High	82 (30.15)	116 (42.03)	2.08 (1.12–3.88)	0.02
Very high	0	4 (1.45)		
**Caregiver variables**				
**Age**	31.56 (0.44)	28.80 (0.39)	0.94 (0.91–0.96)	<0.001
**Sex (female)**	272 (100)	277 (99.28)		0.99
**Education level**				
Illiterate	43 (15.93)	39 (14.66)	References	
Primary	90 (33.33)	95 (35.71)	1.16 (0.69–1.95)	0.56
Secondary	33 (12.22)	42 (15.79)	1.40 (0.74–2.63)	0.29
High school	29 (10.74)	13 (4.89)	0.49 (0.22–1.08)	0. 07
Diploma	56 (20.74)	60 (22.56)	1.18 (0.67–2.08)	0.56
College	19 (7.04)	17 (6.37)	0.98 (0.45–2.16)	0.65
**Outdoor time**	1.46 (0.11)	1.97 (0.13)	1.13 (1.03–1.24)	0.005
**Subscales of Type A personality**				
*TA1*	9.93 (0.25)	10.21 (0.28)	1.01 (0.97–1.05)	0.47
*TA2*	2.59 (0.13)	3.19 (0.14)	1.12 (1.04–1.21)	0.003
**Economic status**				
SES (poorest)	71 (27.5)	33 (13.6)		
SES (second poorest)	51 (19.8)	46 (18.9)	1.94 (1.09–3.44)	0.024
SES (middle)	51 (19.8)	49 (20.2)	2.06 (1.17–3.65)	0.012
SES (second richest)	40 (15.5)	60 (24.7)	3.22 (1.81–5.73)	<0.001
SES (richest)	45 (17.4)	55 (22.6)	2.63 (1.48–4.65)	0.001

^a^ *p*.value ≤ 0.05.

**Table 3:** Multivariate analysis of child and caregivers’ psychological factors.

	OR (95%CI)	*p*-value^c^
**Total ADHD**^a^		
Inattentive	5.65 (2.53–12.61)	<0.001
Hyperactive	2.53 (1.65–3.87)	<0.001
**Personality type**^b^		
TA1	1.01 (0.97–1.06)	0.46
TA2	1.10 (1.01–1.20)	0.03

^a^ adjusted for sex, child's age, economic status and **caregiver's Type A personality.**

^b^ adjusted for caregiver's age, economic status and **child's Inattention and Hyperactivity (subscales of ADHD).**

^c^ *p*.value ≤ 0.05.

## Discussion

The present study found a significant association between childhood burn injuries and caregiver characteristics, including age, time spent outdoors and Type A behaviour. These findings support previous research that has found more burns injuries occur among children with younger caregivers. For example, Shah et al. [27] found that the maternal age of the caregiver was significantly related to the odds of a burn injury. In particular, compared to mothers younger than 20 years old, the odds of childhood burn injury were 40% lower in mothers aged 20–39 years and 72% lower in mothers older than 40 years. In contrast, there has been one study which found that maternal age was higher in the cases than in the controls, but this finding was not statistically significant [28].

A significant association was not found between the caregiver's level of education and childhood burn injuries, although children with high school educated caregivers had lower odds of a burn injury than children with illiterate caregivers. This is in partial agreement with a study in Peru, which found that low maternal education was significantly associated with the odds of childhood burn injury [29]. This association was also previously noted in another longitudinal study on 116 children aged 1–3 years, which reported that the proportion of severe burns was higher among children with less educated mothers [30]. Conversely, a national study in Canada found a higher risk of injury among school aged children whose mothers had an education beyond that of high school [31]. This finding was attributed to the fact that well-educated mothers may be more likely, than less-educated mothers, to report minor events as injuries. Another possible explanation may be that well-educated mothers work more or are involved in more other endeavours, both of which may detract from child supervision.

Childhood injury in general has also been found to be significantly related to a lack of parental supervision, especially for some injuries (e.g. bath drownings). After controlling for confounding variables, such as maternal social support, a study in the United States found that children with tighter maternal supervision had lower odds of childhood injuries [32]. However, the presence of caregiver supervision does not fully account for burn injuries among children [33]. A study in Taiwan found that 78% of burns occurred under the supervision of one or both parents. Clearly more precise research is needed to clarify the relationship between supervision and child burn injuries, particularly in relation to the quality of supervision and its dimensions [33]. A systematic review on the relationship between parent/caregiver supervision and child injuries noted that identifying a direct link between the dimensions of supervision (including attention, proximity, and continuity) and child injury risk was uncommon. In the present study, the amount of time the caregiver spent outdoors was found to be positively associated with the odds of a burn injury, which may be related to the caregiver's level of supervision. Future studies must consider all three dimensions of supervision in order to provide a more complete understanding of the relationship between supervision and injury [34].

Our findings also suggest that the higher the socio-economic status of the household the more likely the child will experience a burn injury. This issue is controversial in the literature. While some studies have reported that children with moderate socio-economic status had higher odds of injuries, than those of low socio-economic status [35], other research has found a significant positive association between low socio-economic status and burn injuries [29, 36]. One possible explanation for these conflicting findings is the dissimilar methods used for measuring socio-economic status. Some methods of assessing socio-economic status use expenditure as an indicator of economic status. This method may generate an incorrect finding due to inaccuracies in recalling details, or an unwillingness to disclose certain types of spending [37]. At the time the present study was initiated there was no validated measure of Iranian socio-economic status available. Therefore, the investigators used their own approach to assessing socio-economic status. Hence, the expenditure capacity assessed in this study may not be exactly the same as socio-economic status. Moreover, in the determination of socio-economic status other aspects should be taken into account.

Independent of the caregiver's age, socio-economic status and *TA2* were found to be positively associated with childhood burn injuries, as were the child's scores on the *Inattention* and *Hyperactivity* measures of ADHD. This association was previously reported in a Chinese adult population [38]. According to that study, people with Type A behaviour pattern had higher odds of injuring themselves as well as their children. Previous research has investigated the relationship the caregivers psychological factors have with burn injuries among children. For example, a study in the USA found that the odds of having injured children was higher among overwhelmed mothers than among emotionally stable mothers [39]. This study also indicated that the odds of child burns increased when his/her mother was tired [39]. Furthermore, both of these variables remained significant, even after adjusting for maternal education and the child's behaviour. However, to the best of our knowledge this is the first study to find a link between the caregiver’s Type A behaviour and childhood injuries.

Investigators have also hypothesized that parental psychopathology may increase a child’s burn risk, but this issue has not been empirically evaluated. Nonetheless, a UK study found that around 33% of burns occurred in families which had experienced recent emotional upset from a major life event. However, no comparison group was included in that study to assess whether or not this degree of emotional upset also existed among families with uninjured children [40].

Another important finding from the present study was that the odds of burn injuries increased by 2.1 times and 1.7 times for each 10 point increment in the *Inattentive* and *Hyperactive* subscales of ADHD, respectively. This finding confirms that the *Inattentive* subscale is much more important than the *Hyperactive* subscale, with regards to increasing the odds of a burn injury. The multivariate analysis also found that the association between the ADHD subscales and the odds of burn injury were stronger after adjusting for the child's sex and the caregiver's age, socio-economic status and Type A behaviour, suggesting that these variables may negatively confound the association between the ADHD subscales and the odds of a burn injury.

Clearly selection bias and recall bias are two major threats to every case-control study. In the current study three main principals were adhered to in order to minimize the potential for such bias, as recommended by Watcholder. In order to minimize recall bias, the controls were selected to be patients of a similar morbidity level, rather than using healthy controls. Although this will not prevent recall limitation, it will minimize recall bias, and complies with the comparable accuracy principle in case-control studies. In order to minimize selection bias the common source population principal was also adhered to. Specifically, controls were selected from a specific paediatric hospital known to have similar referral levels, ensuring a substantially identical source population [24, 25, 41].

Despite a moderate sample size and 12 month data collection period, the study was not large enough to conduct a subgroup analysis for the outcome including scalds, flame, and contact burns; or to conduct subgroup analysis for the predictors, such as gender and age group. Nevertheless, this does not compromise the main aim of study and provides better generalizability for the total population and general prevention programs. Furthermore, measuring and addressing a wide range of possible burn injury predictors is other strength of this study.

## Conclusion

This study found that Type A behaviour, being younger, time spent outdoors, and high economic status were caregiver predictors of burn injuries among Iranian children. Moreover, the association between a child's ADHD score and the odds of a burn injury may be negatively confounded by the characteristics of the caregiver and the child.
